# Phenotypic plasticity vs. local genetic adaptation: essential oil diversity of natural immortelle (*Helichrysum italicum* (Roth.) G.Don) populations along eastern Adriatic coast

**DOI:** 10.3389/fpls.2025.1467421

**Published:** 2025-02-05

**Authors:** Tonka Ninčević Runjić, Dejan Pljevljakušić, Marko Runjić, Martina Grdiša, Zlatko Šatović

**Affiliations:** ^1^ Department of Plant Sciences, Institute for Adriatic Crops and Karst Reclamation, Split, Croatia; ^2^ Institute for Medicinal Plants Research “Dr. Josif Pančić”, Belgrade, Serbia; ^3^ Department of Plant Biodiversity, University of Zagreb, Faculty of Agriculture, Zagreb, Croatia; ^4^ Centre of Excellence for Biodiversity and Molecular Plant Breeding (CoE CroP-BioDiv), Zagreb, Croatia

**Keywords:** *Helichrysum italicum*, field trial, phenotypic plasticity, local adaptation, secondary metabolites, chemodiversity, bioclimatic variables, Mediterranean

## Abstract

The essential oil of *Helichrysum italicum* (Roth) G.Don, commonly known as immortelle, is produced in Mediterranean countries to meet the increasing demand of the cosmetic and pharmaceutical industries. This study focused on the analysis of secondary metabolites, specifically essential oils, extracted from plants grown from the seeds of natural immortelle populations collected along the eastern Adriatic coast and cultivated *ex situ* under uniform conditions. Field trials were conducted to determine whether the observed variability was due to phenotypic plasticity or local genetic adaptation. Eighteen natural immortelle populations were sampled, hydrodistilled and their essential oil composition determined by gas chromatography-mass spectrometry. A total of 84 compounds were identified. Eighteen essential oil compounds were present in concentrations greater than 5% in at least one sample of 18 populations. The populations differed significantly in nine essential oil compounds: Limonene, linalool, nerol, neryl acetate, trans-caryophyllene, neryl propionate, *ar*-curcumene, β-selinene and δ-selinene and the differences were attributed to genetic adaptation to the native environment. Three chemotypes were identified within which the populations were grouped. Results showed a significant and strong correlation between biochemical and bioclimatic distance, with 22.4% of biochemical differentiation between populations explained by bioclimatic distance. Correlations between the 18 main compounds and the bioclimatic variables of the populations’ native environment revealed that BIO14 Precipitation of driest month and BIO15 Precipitation seasonality, were the most informative. These results can serve as a first step for future selection of immortelle populations with desirable adaptations to obtain commercial cultivars that ensure high quality immortelle essential oil.

## 1 Introduction

The synthesis and accumulation of different secondary metabolites varies considerably in the same plant species growing under different environmental conditions. Each metabolite plays an important role in the plant’s response to both biotic and abiotic stress factors. As the effects of environmental change on plant distribution, growth and development intensify, there is an increasing focus on detecting phenotypic variation through responses to phenotypic plasticity or local genetic adaptation (Merilä and Hendry, 2014; MacLean and Beissinger, 2017). Phenotypic plasticity and local adaptation serve as pivotal strategies for organisms to cope with fluctuating environmental conditions. These mechanisms manifest differently depending on population dynamics and the selective pressures they encounter. Phenotypic plasticity, defined as a capacity of a single genotype to exhibit different phenotypes when exposed to diverse environmental conditions, enables the organisms to adjust to environmental shifts. This dynamic response facilitates survival and reproduction under different environmental conditions and provides organisms with a flexible strategy to thrive amid changing landscapes (Via et al., 1995; Pigliucci, 2001; Chevin and Hoffmann, 2017). In contrast, local adaptation involves genetic changes leading to enduring phenotypic traits and increased fitness in their native habitats. These adaptations usually persist over generations, shape the genetic makeup of populations and enable long-term survival in their respective ecological niches (Kawecki and Ebert, 2004; Fan et al., 2016).

In order to investigate phenotypic plasticity in plant species, plants of identical genetic background are exposed to different environmental conditions in either controlled laboratory or field trials. When phenotypic plasticity of outcrossing wild plant species is in question, the investigation based on representative seeds collected from the entire population can provide a more comprehensive understanding. This approach captures the genetic diversity present in the population and reveals how different genetic backgrounds respond to environmental factors. Essentially, it highlights how different environmental conditions impact the species as a whole (Draghi and Whitlock, 2012). Using seeds from individual plants allows for more precise control of genetic variation, which is important for research focused on uncovering the genetic basis of phenotypic plasticity. However, this approach may overlook the broader context of population-level plasticity and may not be appropriate for addressing ecological or comparative questions in phenotypic studies (Gianoli and Valladares, 2012).

If local genetic adaptation is to be investigated, the analysis of plant traits should be carried out on populations grown *ex situ* under uniform conditions rather than *in situ* at sampling sites. In this way, the influence of environmental conditions at the sampling site on plant traits (including secondary metabolites) is reduced (Kawecki and Ebert, 2004; Anderson et al., 2011; Savolainen et al., 2013; de Villemereuil et al., 2022).

Medicinal and aromatic plants play an important role in society; they represent a large group of plants with considerable economic importance (Pandey et al., 2019). Their use is wide-ranging, as they are used in many industrial sectors: medicine, veterinary medicine, food and cosmetics. Over the last 20 years, the demand for plant compounds, essential oils, aromatic chemicals and pharmaceuticals has increased on the global market. However, the mechanisms that influence their synthesis are not yet fully understood (Bakkali et al., 2008). The diversity of secondary metabolites produced by plants at various organizational levels, from individual organs to large communities is referred to as plant chemodiversity. It can be assessed both within and across units of scale; it includes the number of secondary metabolites per individual and the variations in secondary metabolites among individuals within a population, their concentration, structural, and functional variability (Wetzel and Whitehead, 2020). Chemodiversity is shaped by a number of abiotic and biotic factors as well as genetic influences (Kessler and Kalske, 2018). When individuals within a species vary in the chemical composition within metabolite classes, they are often categorized into different chemotypes, which are often used in context of essential oils (Clarke, 2008; Tisserand and Young, 2014). Essential oils have become of interest to both industry and scientific research due to their versatile biological properties (Bakkali et al., 2008) and various applications (Clarke, 2008; Tisserand and Young, 2014).

A high chemical diversity of populations is common in Mediterranean medicinal and aromatic plants such as sage (Jug-Dujaković et al., 2012), rosemary (Ben Abada et al., 2020), oregano (Stešević et al., 2018; Bakha et al., 2020) and thyme (Etri and Pluhár, 2024).

Another important Mediterranean plant species best known for its essential oil diversity is immortelle (*Helichrysum italicum* (Roth) G.Don; Asteraceae), a perennial semi-shrub with bright yellow inflorescences. It is widespread in the Mediterranean region from sea level to 2200 m a.s.l (Galbany-Casals et al., 2011). *H. italicum* is divided into four subspecies namely *H. italicum* ssp. *italicum*; *H. italicum* ssp. *microphyllum*; *H. italicum* ssp. *siculum*, and *H. italicum* ssp. *tyrrhenicum*, among which ssp. *italicum* is the most widespread (Herrando-Moraira et al., 2016). *H. italicum* is an outcrossing, entomophilous and anemochorous species (Galbany-Casals et al., 2011). Immortelle essential oil is located in glandular hairs on flower petals, sepals, bracts, and stem leaves (Perrini et al., 2009). A wide range of variations have been identified in the composition of the essential oil of immortelle (Angioni et al., 2003; Paolini et al., 2006; Mancini et al., 2011; Maksimovic et al., 2013; Talic et al., 2021). Numerous scientific studies, which are summarized in (Ninčević et al., 2019), confirm the health-promoting properties of immortelle. Kramberger et al. (2021) reviewed the literature and concluded that immortelle is effective and safe for internal use. It is used in the cosmetic, pharmaceutical and food industries for its anti-inflammatory (Sala et al., 2002), antioxidant (Kladar et al., 2015; Dzamic et al., 2019; Talić et al., 2019), antimicrobial (Mekinić et al., 2016; Djihane et al., 2017; Staver et al., 2018; Balázs et al., 2022), antiviral activity (Mucsi et al., 1992; Appendino et al., 2007), and anti-cancer compounds (Gismondi et al., 2020).

Croatia has a large production of essential oil using immortelle, which grows naturally on the eastern Adriatic coast and on the islands. Researchers who studied the chemical composition of *H. italicum* natural populations found great variability in the composition of the essential oil as above mentioned. This diversity is attributed to the influence of geographical origin (Morone-Fortunato et al., 2010), environmental factors (Bianchini et al., 2009; Melito et al., 2016), growth stage (Blazevic et al., 1995; Talić et al., 2019); plant parts (Kunc et al., 2022), genotype (Mastelic et al., 2005; Ninčević et al., 2021) and processing methods (Jažo et al., 2022). The majority of the studies analyzing *H. italicum* essential oils composition were performed on wild immortelle populations collected *in situ* or in commercial plantations (with more or less unknown origin of the plant material). None of the studies aimed to resolve whether the diversity of immortelle essential oils is environmentally induced or the result of local genetic adaptation. To clarify the latter, in this study, field trials were conducted that allowed us to clarify the main causes of intraspecific variability in immortelle essential oil. We have sampled 18 natural populations of *H. italicum*, a model plant species for studying adaptation as it grows along the eastern Adriatic coast and islands along the northwest-southeast environmental gradient. By conducting field trials using population as a study unit, we aimed to (1) evaluate the variability in essential oil composition; (2) clarify whether this variability is due to phenotypic plasticity or local adaptation, and; (3) identify the environmental factors at sampling sites that contribute most to the variability obtained.

## 2 Materials and methods

### 2.1 Sampling and plant material

**Figure 1:**
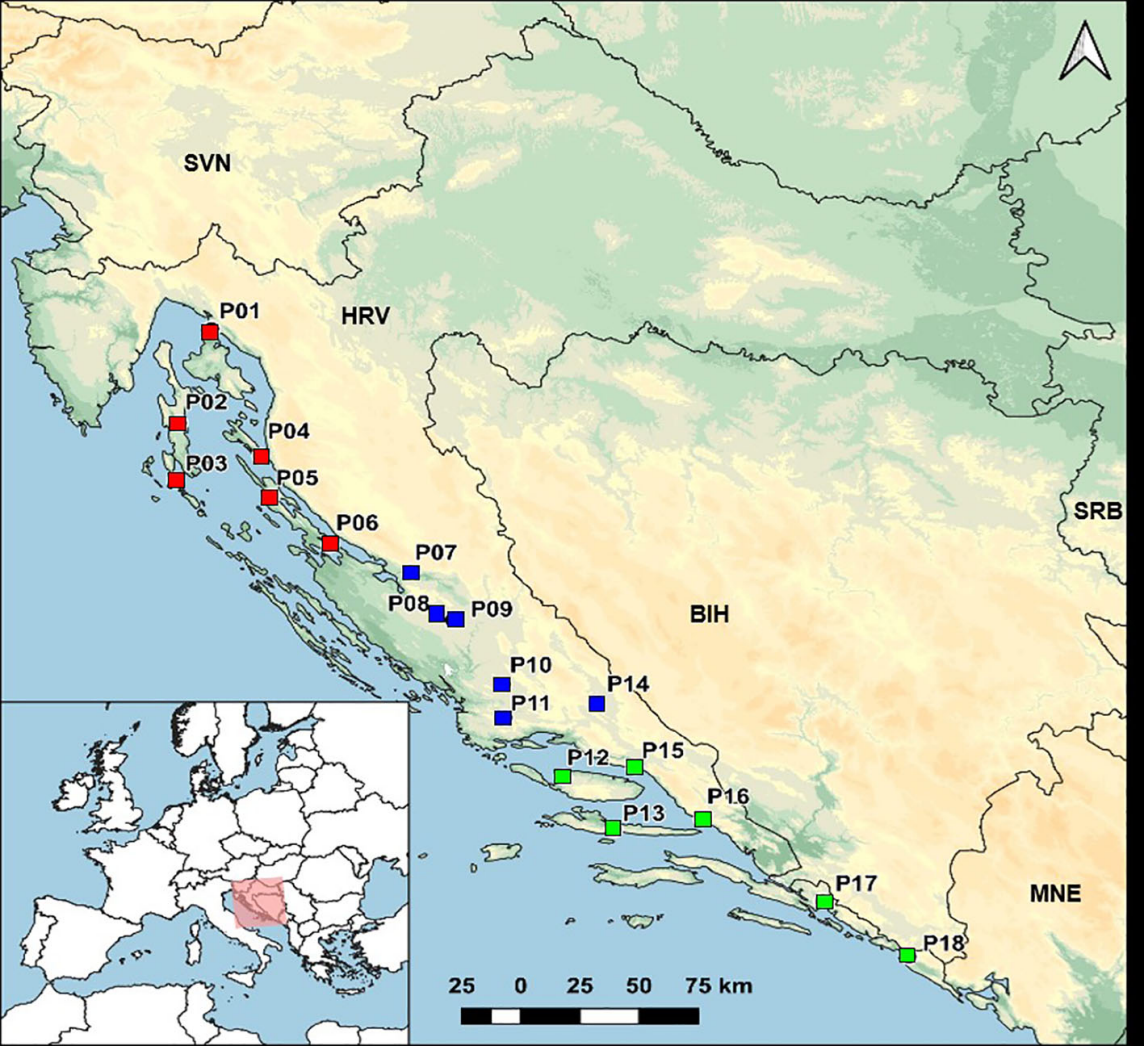
Sampling sites of 18 natural populations of *Helichrysum italicum*. Red squares represent northern Adriatic populations, blue squares represent central Adriatic populations, and green squares represent southern Adriatic populations.

Seed samples from eighteen natural populations of *H. italicum* were collected in three geographical regions (northern, central and southern Adriatic) covering the native distribution range of immortelle along the eastern Adriatic coast in Croatia (**Table 1**; **Figure 1**). The seeds further used in the field experiments were randomly sampled from individuals within each population (ca. 25 individuals, one flower head per individual). Seed samples from all populations are stored in the Collection of Medicinal and Aromatic Plants at the University of Zagreb, Faculty of Agriculture under the accession numbers MAP02672-MAP02689 (data available at the Croatian Plant Genetic Resources Database; https://cpgrd.hapih.hr/). Voucher specimens are identified and deposited in the ZAGR Virtual Herbarium, Zagreb, Croatia (available at: http://herbarium.agr.hr/; Herbarium IDs: 38977, 38981, 38983, 44230–44237, 59856–59862).

**Table 1:** Analysis of variance of the 18 most abundant essential oil compounds of *Helichrysum italicum* populations: the significance of the sources of variability and the average values (%) of the populations.

Source of variability		C03	C13	C21	C35	C42	C48	C49	C50	C51	C56	C57	C58	C59	C62	C67	C72	C73	C80
Population	P(F)	ns	**	***	***	***	*	ns	ns	***	ns	***	***	*	ns	ns	ns	ns	ns
Location	P(F)	ns	ns	ns	ns	ns	ns	ns	ns	ns	ns	ns	ns	ns	ns	ns	ns	ns	ns
Population × Location	P(F)	ns	ns	ns	ns	ns	ns	ns	ns	*	ns	ns	ns	ns	ns	ns	ns	ns	ns
Replicate (Location)	P(F)	ns	ns	ns	ns	ns	ns	ns	ns	ns	***	ns	ns	ns	ns	ns	*	*	ns
Population	P01	4.40 ^ab^	2.69^ab^	3.71^abc^	4.06 ^a^	21.17 ^abc^	4.02 ^a^	0.83 ^a^	4.12 ^a^	5.28^abc^	2.19^a^	6.08^ef^	6.95^f^	3.66^a^	0.07 ^ab^	3.37^a^	3.16^a^	0.20^a^	3.01^a^
	P02	7.19 ^ab^	2.64 ^ab^	4.22 ^a^	3.91 ^a^	21.16 ^abc^	4.49 ^a^	0.89 ^a^	4.57 ^a^	5.70 ^ab^	3.01^a^	4.98^f^	6.39^f^	3.06^a^	0.18 ^ab^	3.53^a^	2.21^a^	0.23^a^	2.16^a^
	P03	3.56 ^ab^	1.29 ^b^	3.05 ^abc^	4.19 ^a^	29.21^a^	3.92 ^a^	0.53 ^a^	5.72 ^a^	6.22 ^a^	3.36^a^	4.85^f^	6.36^f^	2.49^a^	0.18 ^ab^	4.76^a^	0.97^a^	0.22^a^	1.83^a^
	P04	4.80 ^ab^	3.18 ^ab^	2.40 ^abc^	3.30 ^ab^	26.33 ^ab^	2.19 ^a^	1.91 ^a^	4.20 ^a^	4.28^abcd^	2.31^a^	6.45^def^	6.05^f^	3.02^a^	0.27 ^ab^	3.35^a^	2.38^a^	0.43^a^	2.46^a^
	P05	7.74 ^ab^	2.09 ^b^	1.37 ^c^	2.76 ^abcd^	22.22 ^abc^	3.67 ^a^	2.32 ^a^	2.77 ^a^	1.57 ^de^	3.23^a^	8.02^cdef^	6.37^f^	2.96^a^	0.36 ^ab^	2.45^a^	4.43^a^	0.40^a^	3.74^a^
	P06	3.75 ^ab^	3.09 ^ab^	2.14 ^abc^	2.42 ^abcde^	18.94 ^abcde^	3.77 ^a^	1.89 ^a^	4.29 ^a^	2.43 ^cde^	1.88^a^	9.11^bcdef^	9.83^bcdef^	4.64^a^	0.34 ^ab^	4.23^a^	3.60^a^	0.64^a^	3.25^a^
	P07	1.88 ^b^	1.65 ^b^	1.81 ^abc^	1.82 ^bcde^	15.87 ^abcde^	3.27 ^a^	0.73 ^a^	8.49 ^a^	2.73^bcde^	1.67^a^	9.69^abcdef^	8.87^cdef^	5.23^a^	1.96 ^a^	2.66^a^	2.80^a^	0.50^a^	1.61^a^
	P08	3.05 ^ab^	1.89 ^b^	2.05 ^abc^	3.36 ^ab^	16.09 ^abcde^	2.76 ^a^	2.11 ^a^	7.43 ^a^	2.18 ^de^	2.34^a^	11.70^abcde^	8.60^cdef^	3.41^a^	0.45 ^ab^	3.64^a^	2.71^a^	0.38^a^	2.64^a^
	P09	8.52 ^ab^	3.23 ^ab^	1.76 ^bc^	2.73 ^abcd^	18.67 ^abcde^	3.86 ^a^	0.76 ^a^	4.02 ^a^	2.81^bcde^	4.22^a^	10.25^abcdef^	6.91^f^	2.72^a^	0.04 ^ab^	2.54^a^	1.71^a^	0.27^a^	2.33^a^
	P10	5.80 ^ab^	2.11 ^b^	2.65 ^abc^	2.76 ^abcd^	15.43 ^abcde^	4.50 ^a^	0.58 ^a^	6.42 ^a^	2.91^bcde^	3.57^a^	9.62^abcdef^	8.60^cdef^	3.51^a^	0.41 ^ab^	2.23^a^	1.40^a^	0.90^a^	3.42^a^
	P11	6.10 ^ab^	1.58 ^b^	2.88 ^abc^	3.11 ^abc^	20.68 ^abcd^	4.80 ^a^	0.58 ^a^	7.33 ^a^	3.21^abcde^	2.48^a^	10.01^abcdef^	7.18^ef^	2.49^a^	0.00 ^b^	3.85^a^	1.34^a^	0.90^a^	2.50^a^
	P12	6.39 ^ab^	2.36 ^b^	1.59 ^c^	1.02 ^de^	7.09 ^cde^	3.77 ^a^	3.10 ^a^	6.85 ^a^	1.37 ^de^	2.17^a^	14.71^ab^	14.75^a^	5.64^a^	0.67 ^ab^	0.48^a^	3.05^a^	0.45^a^	3.25^a^
	P13	5.82 ^ab^	1.80 ^b^	4.07 ^ab^	1.15 ^de^	5.66 ^de^	5.44 ^a^	0.83 ^a^	7.36 ^a^	2.58 ^cde^	2.75^a^	12.74^abcd^	11.96^abcd^	5.26^a^	0.19 ^ab^	0.58^a^	2.81^a^	0.50^a^	3.12^a^
	P14	5.70 ^ab^	2.54 ^ab^	2.09 ^abc^	3.42 ^ab^	16.32 ^abcde^	3.55 ^a^	1.01 ^a^	6.49 ^a^	2.91 ^bcde^	3.80^a^	6.78^def^	7.30^def^	3.17^a^	0.27 ^ab^	2.99^a^	3.75^a^	0.85^a^	3.97^a^
	P15	5.85 ^ab^	5.50 ^a^	2.12 ^abc^	1.32 ^cde^	12.17 ^bcde^	4.24 ^a^	0.80 ^a^	8.13 ^a^	1.66 ^de^	1.79^a^	9.35^abcdef^	11.87^abcde^	5.61^a^	0.24 ^ab^	2.15^a^	2.14^a^	2.51^a^	3.85^a^
	P16	10.55 ^a^	3.97 ^ab^	1.37 ^c^	1.21 ^cde^	12.55 ^bcde^	5.19 ^a^	3.11 ^a^	3.84 ^a^	1.21 ^e^	2.16^a^	11.72^abcde^	9.01^cdef^	3.75^a^	0.33 ^ab^	1.30^a^	1.99^a^	0.36^a^	2.52^a^
	P17	6.95 ^ab^	1.87 ^b^	2.38 ^abc^	1.39 ^cde^	7.05 ^cde^	5.37 ^a^	0.64 ^a^	7.73 ^a^	3.06 ^bcde^	2.23^a^	13.38^abc^	14.01^ab^	5.29^a^	0.23 ^ab^	3.34^a^	1.74^a^	1.26^a^	2.44^a^
	P18	5.71 ^ab^	1.34 ^b^	1.59 ^c^	0.79 ^e^	5.38 ^e^	2.81 ^a^	2.22 ^a^	8.98 ^a^	1.24 ^de^	1.99^a^	15.76^a^	12.46^abc^	4.12^a^	0.74 ^ab^	0.72^a^	3.47^a^	2.92^a^	2.35^a^
Average	5.77	2.49	2.40	2.48	16.22	3.98	1.38	6.04	2.96	2.62	9.73	9.08	3.89^a^	0.39	2.68	2.54^a^	0.77	2.80	

P(F) - significance of the ANOVA’s F-test (significance level: ***P < 0.001, **0.001 < P < 0.01, *0.01 < P < 0.05, ^ns^P > 0.05); Average values (%) of the populations labelled with the same letter are not significantly different at the P > 0.05 level according to Tukey’s test.

### 2.2 Field trial

Immortelle seedlings were grown in a greenhouse at the Institute for Adriatic Crops and Karst Reclamation in Split and planted at two different field trial locations: at the Institute in Split (43°30’18.23.”N, 16°29’59.51”E) and in Vojnić Sinjski (43°36’00.19.”N, 16°42’22.77”E). These field trial sites were selected because their ecological conditions represent two typical natural conditions under which immortelle is grown commercially. The field trial at the Institute in Split (1) represents a typical coastal habitat with a moderately warm humid climate with hot summers (Cfa) according to the Köppen classification (Filipčić, 2000). At this location, the soil is rendzic leptosol developed on flysch depositions, with high carbonate content, alkaline reaction, low organic matter content, and clay-loamy texture. The experimental field in Vojnić Sinjski (2) represents a hinterland with a moderately warm humid climate with warm summers (Cfb) (Filipčić, 2000). The soil is calcic cambisol developed on limestone, with neutral reaction, moderate organic matter content, and clay-loamy texture.

The field trials were set up as randomized complete block designs (RCBD) with two replicates in rows 0.8 m apart with 0.4 m between plants. Each of the 18 populations was represented by 20 plants in two replicates. Fertilizers, irrigation, and plant protection measures were not applied.

The immortelle inflorescences (with the stem and leaves) were harvested in the full flowering phenophase. The samples were air-dried at room temperature. A total of 72 samples were analyzed (18 populations × 2 field trial locations × 2 replicates).

The same 18 immortelle populations were genotyped with AFLP markers to assess genetic diversity and population structure. Results are published in (Ninčević et al., 2021).

### 2.3 Essential oil distillation and analyses

Essential oil was isolated from 100 g of dry plant material per sample by hydrodistillation in a Clevenger-type apparatus for two and a half hours. Due to the very small amount of essential oil (e.g. 0.1%) pentane (1 mL) was added, in the burette of the apparatus, as an essential oil-capturing solvent. The oil samples were dried over with anhydrous sodium sulfate.

Quantitative and qualitative analysis of the essential oil was carried out using Gas chromatography with flame ionization detection (GC-FID) and Gas chromatography-mass spectrometry (GC-MS) (in the laboratory of the Institute for Medicinal Plants Research “Dr. Josif Pančić” in Belgrade, Serbia). Quantitative analysis was performed on model HP-5890 Series II (Hawlett-Packard, Waldbronn, Germany) equipped with the split-splitless injector, HP-5 capillary column (25 m × 0.32 mm, film thickness 0.52 μm) and Flame ionization detector (FID). Hydrogen (1 ml/min) was used as the gas carrier. The injector was heated to 250°C and the detector to 300°C, while the column temperature was linearly programmed from 40 - 260°C (temperature increase 4° C/min). Chromatogram processing results, made as a percentage of the surface area of each identified compound against the total peak area, were used as a basis for quantification. Gas chromatography/mass spectrometry analysis was performed under almost equal analytical conditions, using the HP G 1800C Series II GCD analytical system Hawlett-Packard, (Palo Alto, CA, USA) equipped with HP-5MS column (30 mx 0.25 mm x 0.25 μm). Helium was used as the carrier gas. The transfer line (MSD) was heated to 260°C. The EI mass spectrum (70 eV) is set to scan in m/z mode 40-400. The volume of 1 μL of sample dissolved in ethanol (20 μl/2 ml), injected in split mode (1:30) was analyzed.

Identification of compounds was performed by comparing their mass spectra and retention indices with spectra obtained from authentic samples and/or with NIST/Wiley databases, using different search engines (PBM/NIST/AMDIS) and available literature data (Hochmuth, 2006; Adams, 2007). The percentage of compounds was calculated from electronic measurements using Flame-ionizing detection (FID; 250°C).

### 2.4 Data analysis

Correlations between the 18 major essential oil compounds were calculated based on the Pearson correlation coefficient in SAS v. 9.3 (SAS, 2011). Major compounds were those found in concentrations greater than 5% per sample (18 populations × 2 field trial locations × 2 replicates).

Univariate analyses of variance of the 18 major essential oil compounds were performed using a model including the effects of populations (i.e. sampling sites), field trial locations, and their interaction as fixed variables, and the effect of replicates nested in the field trial locations as a random variable. The model residuals were visually analyzed using Q-Q plots and tested for normal distribution using the Shapiro-Wilk test. Comparisons of means between populations were performed using Tukey’s *post hoc* test at *P* < 0.05. The analysis was performed using the GLM procedure in the SAS software.

Principal component analysis (PCA) was performed on nine essential oil compounds, which showed significant differences (*P* < 0.05) between the populations. A biplot with the populations and the essential oil constituents (as vectors) was constructed using the first two principal components using the PRINCOMP procedure in the SAS.

The Euclidean distance matrix between all population pairs was calculated on the basis of nine essential oil compounds. The matrix was used in the cluster analysis (CA) with the Ward method (Ward, 1963) in the CLUSTER procedure in SAS. The optimal number of clusters was determined based on the value of the pseudo F-statistic (PSF). The populations were therefore classified into clusters representing different chemotypes.

A univariate analysis of variance was performed using the GLM procedure in SAS to test the mean differences between the chemotypes with respect to the content of the nine essential oil compounds. A comparison of means between clusters was performed using Tukey’s *post hoc* test at *P* < 0.05.

The discriminant analysis (DA) was performed with the procedures STEPDISC, DISCRIM and CANDISC in the SAS program. Stepwise discriminant analysis (STEPDISC) allowed the selection of the compounds that contributed most to the classification of the populations into the three identified chemotypes. The success of the classification was determined by cross-validation with the DISCRIM procedure. The CANDISC procedure was used to run the discriminant function and construct a biplot showing the populations and essential oil constituents (as vectors).

Four distance matrices were used to analyze the relationships between geographical, bioclimatic, genetic, and biochemical data. The matrix of the natural logarithm of geographical distances (in km) between population pairs was calculated as described by (Rousset, 1997). Bioclimatic data for 18 sampling locations were obtained from the WorldClim database (www.worldclim.org) and comprised 19 bioclimatic variables (11 temperature-related and eight precipitation-related) representing the annual trends (e.g., mean annual temperature, annual precipitation), seasonal variations (e.g., annual range in temperature and precipitation), and extremes in temperature and precipitation (Hijmans et al., 2005). The bioclimatic distance matrix was constructed using the Euclidean distances between populations based on 19 bioclimatic variables. The matrix of pairwise *F_ST_*/(1 - *F_ST_*) ratios between populations based on previously published AFLP data (Ninčević et al., 2021); see Materials and Methods) was used as the genetic distance matrix, while the biochemical distance was calculated as the Euclidean distance between populations based on nine essential oil components showing significant differences between the populations. Pearson correlation coefficients between the geographical, bioclimatic, genetic and biochemical distances were calculated and significance was assessed using Mantel tests (Mantel, 1967) with 10,000 permutations as implemented in NTSYS-pc v2.21L (Rohlf, 2005). In addition, the correlations between latitude, longitude, each of the 19 bioclimatic variables and the 18 major essential oil compounds were also calculated.

## 3 Results

### 3.1 Variability in essential oil composition

The essential oils of 18 *H. italicum* populations (**Figure 1**; **Supplementary Table S1**) were analyzed, resulting in the detection of 90 compounds, out of which 84 were identified (**Supplementary Table S2**). Notably, 50 of these compounds were found to be present in all 18 *H. italicum* populations. Eighteen essential oil compounds were present in concentrations greater than 5% in at least one sample from 18 populations. They were selected for further data analysis (**Supplementary Table S3**).

Coefficients of variation (*CV*) of 18 essential oil compounds showed high chemical diversity of *H. italicum* populations (**Supplementary Table S3**). Compound β-curcumene (C62) showed the highest degree of variability (198.94%) and β-selinene (C58) the lowest (33.86%) across all samples.

Correlations between the 18 main essential oil compounds were calculated. High levels of intercorrelation among compounds were found as shown in **Supplementary Table S4**. We can conclude that nerol and esters were positively correlated, and negatively with sesquiterpenes selinene and *ar*-curcumene.

The analysis of variance of the 18 most represented essential oil compounds revealed that population (i.e. sampling site) as a source of variation was significant (*P* < 0.05) in the case of nine essential oil compounds, location (i.e. field trial location) had no significant effect on any essential oil compound, while the population × location interaction was significant only in the case of neryl propionate (C51) (**Table 1**).

The populations differed significantly in these nine essential oil compounds: limonene (C13), linalool (C21), nerol (C35), neryl acetate (C42), trans-caryophyllene (C48), neryl propanoate (C51), *ar*-curcumene (C57), β-selinene (C58), and δ-selinene (C59). Neryl acetate was the major compound with a content ranging from 5.38% (P18 Cavtat) to 29.22% (P03 Lošinj) with an average of 16.22%. The highest content of neryl acetate was observed in the northwestern populations. Other major compounds were *ar*-curcumene, which ranged from 4.85% (P03 Lošinj) to 15.77% (P18 Cavtat) with an average of 9.73%, and β-selinene, ranging from 6.05% (P04 Rab) to 14.75% (P12 Brač) with an average of 9.07%. The content of both compounds was higher in the southern immortelle populations. Another major compound was α-pinene, ranging from 1.88% (P07 Obrovac) to 10.55% (P16 Živogošće) (average = 5.77%), but did not differ significantly among populations.

### 3.2 Geographical structuring of the chemotypes

**Figure 2:**
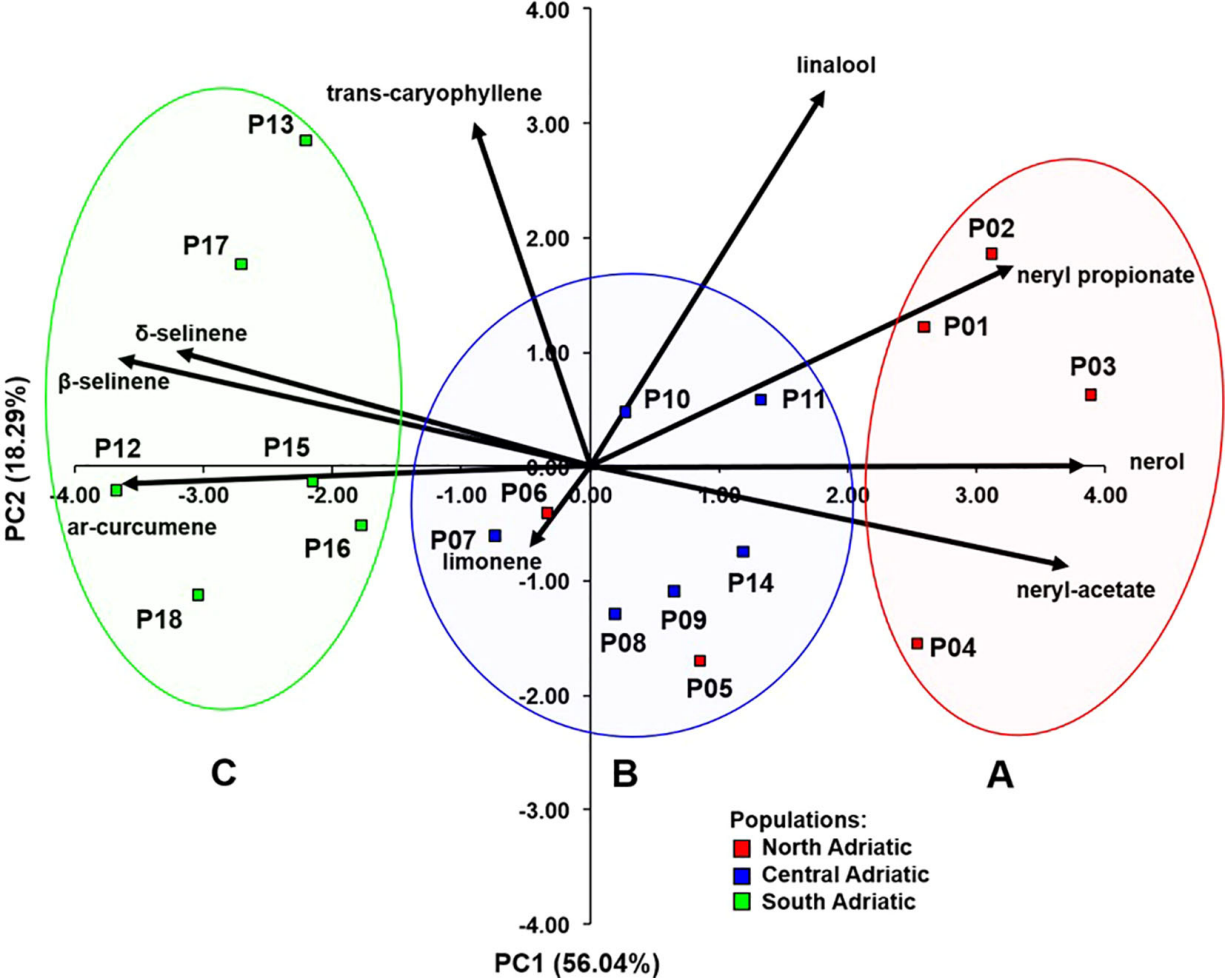
Biplot obtained by principal component analysis based on nine essential oil compounds analyzed in 18 *H. italicum* populations. Clusters **(A–C)** were determined based on cluster analysis.

Nine compounds that showed significant differences between populations were further selected for the principal component analysis (PCA) (**Figure 2**). The first two principal components (PC) had eigenvalues greater than one and together explained 74.33% of the total variability. **Supplementary Table S5**. shows the correlations between the nine compounds of the essential oil and the first three principal components. The first principal component separated northwestern populations (P01 Krk, P02 Cres, P03 Lošinj and P04 Rab; subsequently assigned to chemotype A) rich in nerol (C35), neryl acetate (C42), and neryl propionate (C51) from the southeastern populations (P12 Brač, P13 Hvar, P15 Omiš, P16 Živogošće, P17 Slano and P18 Cavtat; assigned to chemotype C) rich in *ar*-curcumene (C57), β-selinene (C58), and δ-selinene (C59). Central Adriatic populations (P07 Obrovac, P08 Benkovac, P09 Kistanje, P10 Unešić, P11 Seget and P14 Sinj; chemotype B) with two northwestern populations (P05 Zrće, Pag, P06 Miškovići, Pag), showed average values of these compounds and were located around the center of the biplot (**Figure 2**).

**Figure 3:**
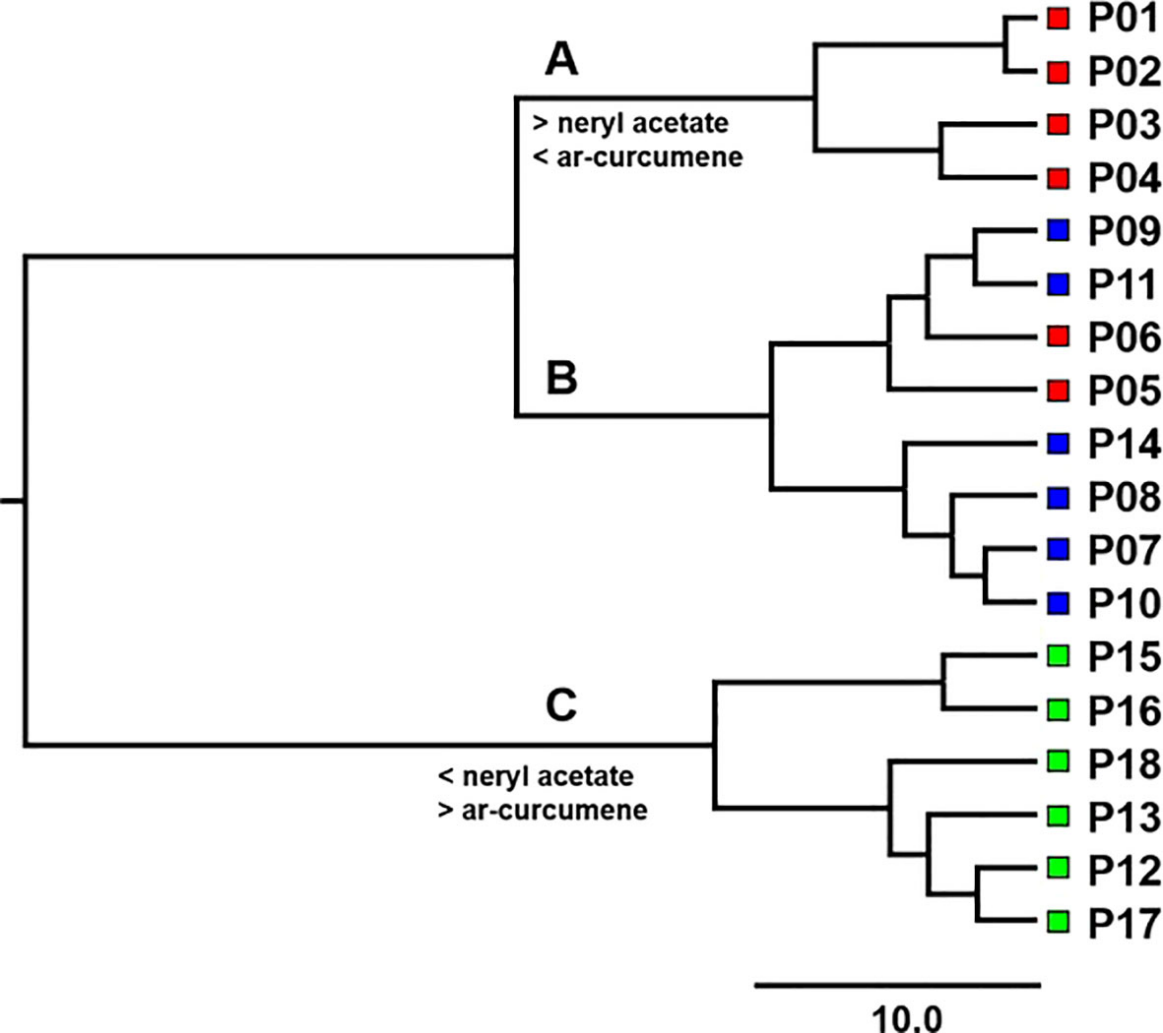
Ward’s dendrogram based on squared Euclidean distances between 18 *H. italicum* populations. The classification of populations into clusters **(A–C)** based on the value of the pseudo F statistics is shown above the branches. Red squares represent northern Adriatic populations, blue squares represent central Adriatic populations, and green squares represent southern Adriatic populations.

Cluster analysis classified populations into three chemotypes as mentioned above. The Euclidean distance between 18 populations was calculated based on nine essential oil compounds (**Supplementary Table S6**). Based on square Euclidean distances between 18 populations of *H. italicum*, Ward’s dendrogram was created (**Figure 3**).

Analysis of variance between clusters revealed that chemotypes differed significantly in seven out of nine main essential oil compounds (*P* < 0.05). The clusters differed the most in the content of nerol (C35), neryl acetate (C42), neryl propionate (C51), *ar*-curcumene (C57), and β-selinene (C58) (**Table 2**).

**Table 2:** Analysis of variance of nine essential oil compounds between chemotypes.

Compound	*P*(F)	Chemotype		
		A	B	C
limonene (C13)	^ns^	2.451^a^	2.273^a^	2.806^a^
linalool (C21)	^*^	3.343^a^	2.092^b^	2.187^ab^
nerol (C35)	^***^	3.866^a^	2.797^b^	1.145^c^
neryl acetate (C42)	^***^	24.469^a^	18.029^b^	8.316^c^
trans-caryophyllene (C48)	^ns^	3.656^a^	3.772^a^	4.469^a^
neryl propianate (C51)	^***^	5.370^a^	2.593^b^	1.852^b^
*ar*-curcumene (C57)	^***^	5.590^c^	9.398^b^	12.945^a^
β-selinene (C58)	^***^	6.439^b^	7.959^b^	12.344^a^
δ-selinene (C59)	^**^	3.059^b^	3.515^b^	4.947^a^

*P*(F) - significance of F-test (***P < 0.001; **0.001 < *P* < 0.01; *0.01 < *P* < 0.05; ^ns^ *P* > 0.05).

Average values of population clusters marked with the same letter are not significantly different at the *P* > 0.05 level.

**Figure 4:**
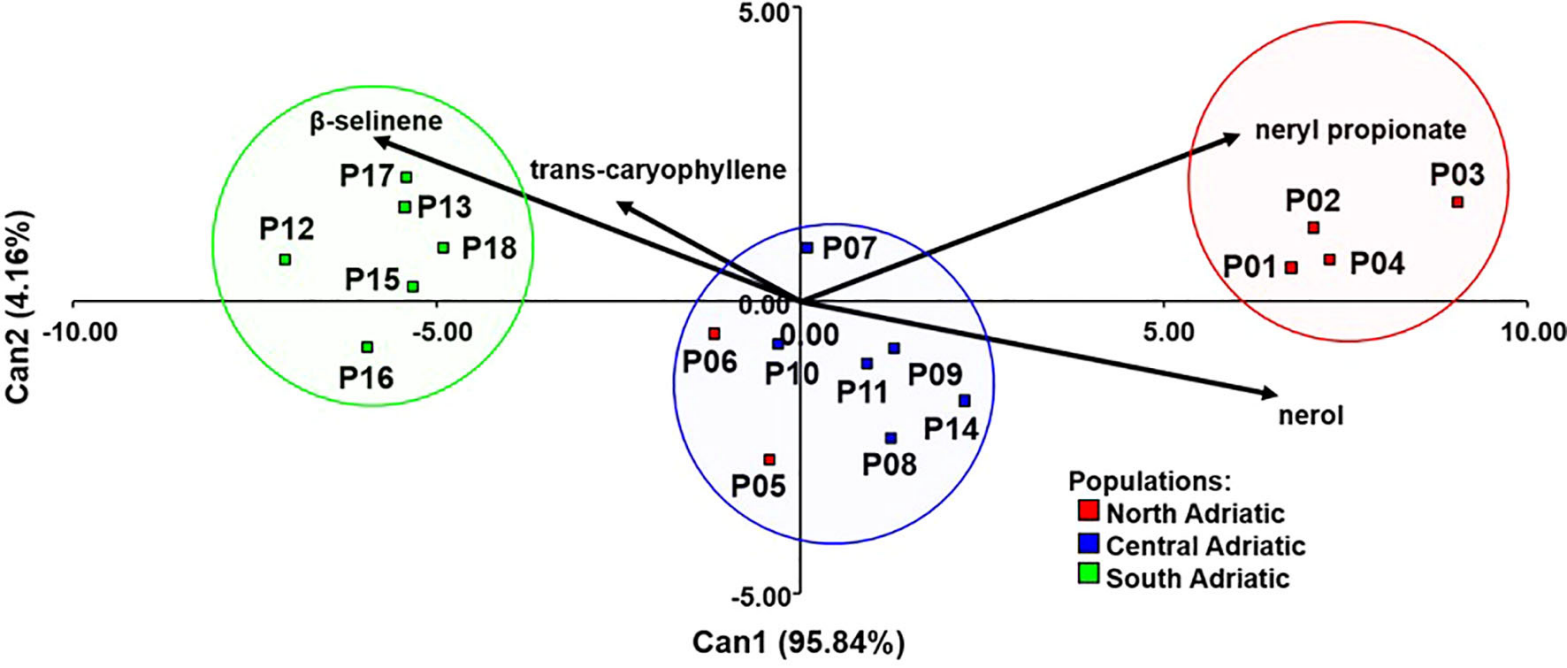
Discriminant analysis of 18 *H. italicum* populations based on four essential oil compounds to distinguish presumed chemotypes.

Based on a stepwise discriminant analysis, the most informative compounds to discriminate the three *H. italicum* chemotypes were nerol (C35), neryl propionate (C51), trans-caryophyllene (C48), and β-selinene (C58) (**Supplementary Table S7**). Based on these compounds, it was possible to correctly classify 100% of the population into presumed chemotypes after the cross-validation procedure (**Figure 4**).

### 3.3 Diversity of essential oil composition as influenced by geography, climate and genetics

**Figure 5:**
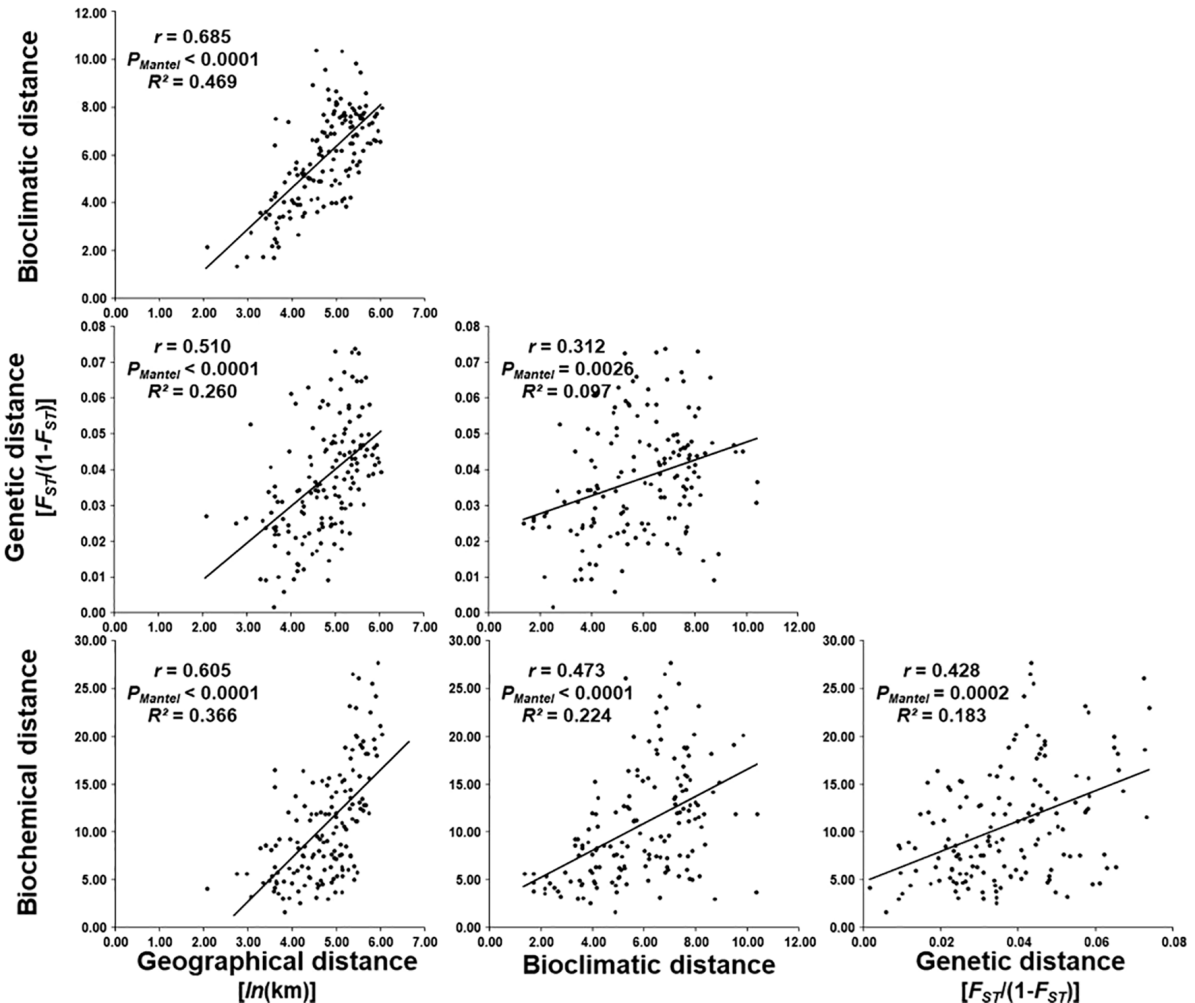
Regression of bioclimatic, genetic [*F_ST_*/(1-*F_ST_*)] and biochemical distance on geographical distance [*ln*(km)], genetic and biochemical distance on bioclimatic distance, and biochemical distance on genetic distance between 18 *H. italicum* populations.

In order to explain the biochemical diversity of the studied *H. italicum* populations, correlations between the geographical, bioclimatic, genetic and biochemical data were analyzed, which is shown in **Figure 5**.

Analysis of the relationship between biochemical and geographical distance revealed that there is a significant correlation (*r* = 0.605, *P_Mantel_* < 0.0001). The coefficient of determination (*R^2^* = 0.366) was obtained, which shows that 36.6% of biochemical diversity can be explained by geographical distance.

Correlation between biochemical and bioclimatic distance was significant and moderate (*r* = 0.473, *P_Mantel_* < 0.0001). Determination coefficient was *R^2^* = 0.224 indicating that 22.4% of biochemical differentiation between analyzed populations can be explained by bioclimatic distance.

Also, a significant correlation was found between biochemical and genetic distance. Correlation between genetic distance and biochemical distance was also significant and moderate (*r* = 0.428; *P_Mantel_* < 0.0002). Determination coefficient was *R^2^* = 0.183 indicating that 18.3% of biochemical differentiation between analyzed populations can be explained by genetic distance.

**Figure 6:**
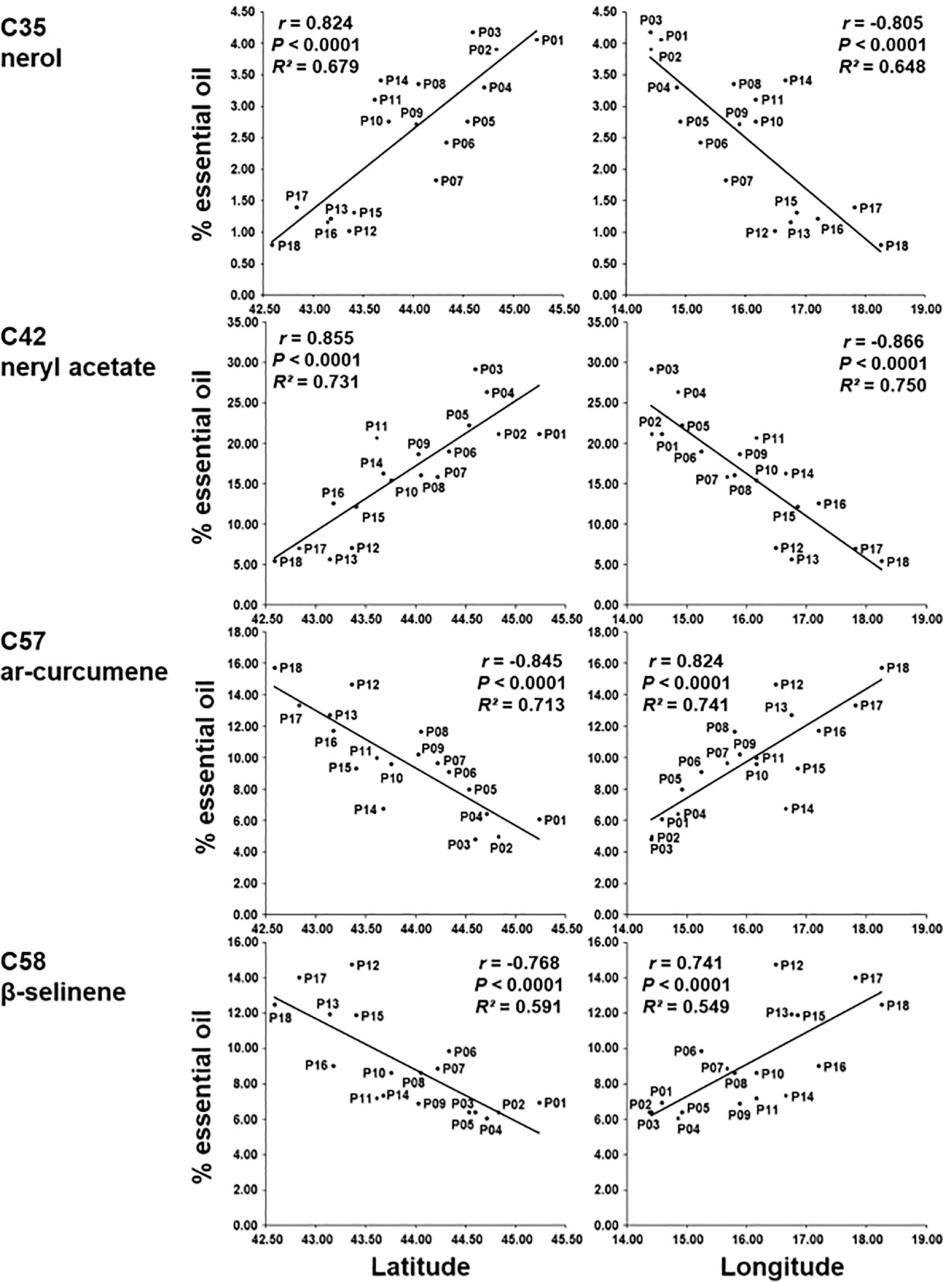
Regression of the percentage of nerol (C35), neryl acetate (C42), *ar*-curcumene (C57) and β-selinene (C58) on latitude and longitude based on 18 *H. italicum* populations.

In addition, correlations between sampling sites (i.e., latitude and longitude) and the 18 major essential oil compounds were also calculated (**Supplementary Table S8**). Four compounds were highly significantly correlated positively or negatively (*r* > 0.70 or *r* < -0.70) with latitude and longitude. Compounds nerol (C35) and neryl acetate (C42) were positively correlated with latitude and negatively correlated with longitude. Compounds *ar*-curcumene (C57) and β-selinene (C58) were negatively correlated with latitude and positively with longitude. The obtained results indicate that the northernmost populations (at the same time and the westernmost) have the most nerol (C35) and neryl acetate (C42), and the southernmost (and easternmost) have the most *ar*-curcumene (C57) and β-selinene (C58) (**Figure 6**).

**Figure 7:**
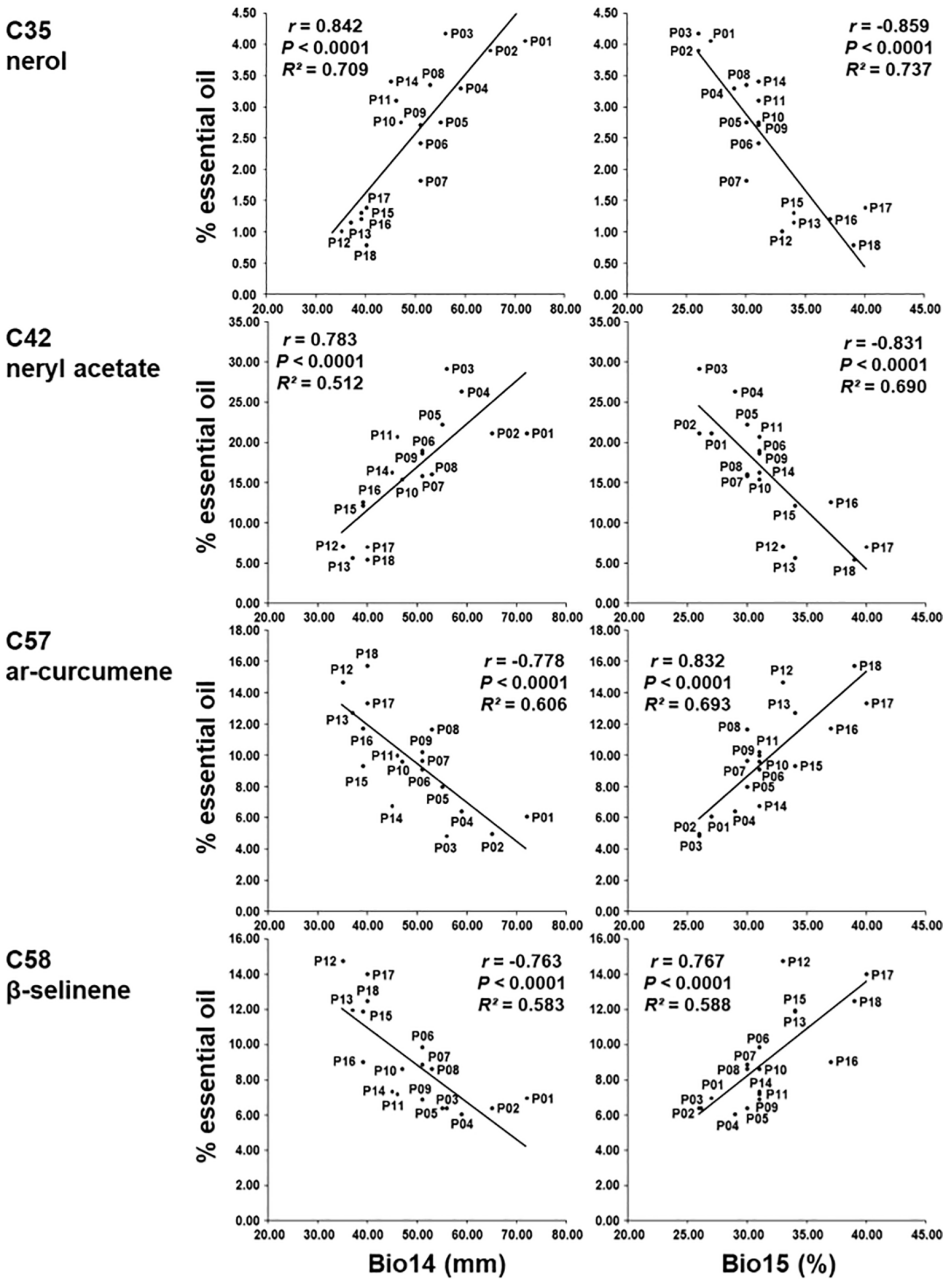
Regression of the percentage of nerol (C35), neryl acetate (C42), *ar*-curcumene (C57) and β-selinene (C58) on BIO14 Precipitation of driest month and BIO15 Precipitation seasonality.

Correlations between each of the 19 bioclimatic variables of the sampling sites and the 18 major essential oil compounds were calculated (**Supplementary Table S9**). Four bioclimatic variables: BIO14 Precipitation of the driest month (measures aridity during the driest month), BIO15 Precipitation seasonality (reflects seasonal variability in rainfall), BIO17 Precipitation of the driest quarter (identifies periods of extreme dryness), and BIO18 Precipitation of warmest quarter (highlights water availability during the hottest part of the year) were the most informative since they had strong correlation (*r* > 0.70) with five essential oil compounds (**Supplementary Table S9**). Compounds nerol (C35), neryl acetate (C42), neryl propanoate (C51) were positively correlated with bioclimatic variables: BIO14 Precipitation of driest month, BIO17 Precipitation of driest quarter and BIO18 Precipitation of warmest quarter and negatively correlated with BIO15 Precipitation seasonality (**Figure 7**) suggesting that a higher proportion of the content of these compounds corresponds to a higher amount of precipitation. Compounds *ar*-curcumene (C57) and β-selinene (C58) were inversely correlated with the above-mentioned variables (negatively correlated with the bioclimatic variables BIO14, BIO17, and BIO18, and positively with BIO15). As the bioclimatic variables BIO14 Precipitation of the driest month, BIO17 Precipitation of driest quarter and BIO18 Precipitation of warmest quarter are very strongly correlated with each other, only BIO14 Precipitation of driest month and BIO15 Precipitation seasonality are shown in **Figure 7**.

## 4 Discussion

### 4.1 Essential oil diversity as a consequence of local genetic adaptation

The physiological and biochemical responses of medicinal and aromatic plants are significantly influenced by many ecologically limiting factors. Plants produce secondary metabolites including essential oils to cope with the negative effects of stress during their growth and development (Pant et al., 2021). These adaptation mechanisms of plants often lead to increased production of various phytochemicals, depending on the environmental factors to which they have been exposed. Previous research has shown that immortelle has a high variability in secondary metabolites (Bianchini et al., 2001; Mastelić et al., 2008; Cávar Zeljković et al., 2015; Oliva et al., 2018; Tzanova et al., 2018), but the main factors influencing the diversity between populations have not been sufficiently investigated. In contrast to numerous studies focusing on essential oils extracted from natural immortelle populations *in situ* or from commercial plantations (with plant material of unknown origin), we conducted field trials *ex situ* with immortelle populations originating from different sampling sites along the eastern Adriatic coast to better understand the possible causes of the diversity of immortelle essential oils. The analysis of essential oils of 18 populations yielded a total of 90 compounds, of which 84 compounds were identified. For comparison, Cávar Zeljković et al. (2015) in their study identified 97 compounds in six immortelle samples, while Mastelić et al. (2008) identified a total of 44 compounds. Kladar et al. (2015) emphasize that although more than 80 compounds in immortelle essential oil have been identified in many scientific studies, many of them are present only in traces and have no major effect on chemotype. The European Pharmacopoeia (European Pharmacopoeia, 2019) does not contain established standards for the main compounds present in the essential oil of *H. italicum*. Therefore, the comparison can only be made with studies by other authors. In our research, 18 essential oil compounds were present in amounts greater than 5% in at least one sample. Neryl acetate was one of the main compounds, which coincides with research of Blazevic et al. (1995), where neryl acetate varied from 4.13 - 13.51%, and research of Mastelić et al. (2008) where the highest concentration of neryl acetate was 10.4%. Other main compounds in our study were *ar*-curcumene, β-selinene α-pinene, italidione I, trans-caryophyllene, neryl propanoate, and γ-curcumene. Compounds α-pinene, α-curcumene, γ-curcumene were also the main compounds in Blazevic et al. (1995), and α-pinene (12.8%) was found in the highest concentration in Mastelić et al. (2008). In research from Ivanovic et al. (2011) from southern part of Croatia (Konavle), compounds γ-curcumene (12.4%), β-selinene (9.9%), trans-β-caryophyllene (6.9%), α-selinene (5.9%), italicene (4.6%) and α-curcumene (4.0%) were the most abundant compounds confirming similarity with our research. Our results partly coincide with the research of Weyerstahl et al. (1986) stating that the essential oils from the former Yugoslavia are characterized by a high content of α-pinene (22%), y-curcumene (10%), β-selinene (6%), neryl acetate (6%) and β-caryophyllene (5%), while along the Adriatic coast the main compounds are α-curcumene (15-29%) or γ-curcumene or α-pinene (25-30%) and neryl acetate (4-14%). The low percentage of α-pinene in the present study could be attributed to the distillation of dry rather than fresh *H. italicum* samples. Similar composition of the essential oil is shown in the study by (Bianchini et al., 2003) on Tuscan *H. italicum* where, depending on the sampling site, the main compounds were α-pinene (33 to 53%) or neryl acetate (10-22%), sesquiterpene hydrocarbons (23-39%) with a significant proportion of β-selinene, β-caryophyllene and α-selinene. The main compounds isolated from *H. italicum* in Montenegro (Kladar et al., 2015) were neryl acetate, γ-curcumene, neryl propionate, and α-curcumene, which partially coincides with the essential oil composition in the present study.

The analysis of variance showed that population, as a source of variation was significant for nine out of 18 essential oil compounds, while field trial location had no significant effect on any essential oil compound. These results suggest that variation in essential oil composition is the result of underlying genetic divergence, particularly exposure and subsequent local genetic adaptation to prevailing environmental conditions, rather than direct adjustment to different environments, known as phenotypic plasticity. A similar observation was made by Zhou et al. (2021a) in the species *Agriophyllum squarrosum* (L.) Moq, where results from the field trial showed that the differences between populations in the accumulation of organic acids could be due to the local adaptation to high altitudes. In contrast, in other study by Zhou et al. (2021b), also on populations of *A. squarrosum*, they found that the accumulation of flavonoids was not related to local adaptation to high altitude. Demasi et al. (2018) investigated peripheral alpine populations of *Lavandula angustifolia* Mill. in a field trial, to observe variation due to genetic adaptation to the native environments and to exclude the short-term response to environmental factors. Their results indicated that ecological conditions of peripheral sites might have induced different adaptation mechanisms in lavender, leading to different phytochemical compositions.

Overall, phenotypic plasticity and local adaptation represent complementary strategies for organisms to navigate environmental challenges. While phenotypic plasticity offers immediate adjustments to varying conditions, local adaptation fosters genetic changes that promote sustained fitness within specific habitats, highlighting the complex interplay between genetic and environmental factors in shaping the evolutionary path of species (Fan et al., 2016; Chevin and Hoffmann, 2017).

### 4.2 Geographical structuring of the chemotypes

Different chemotypes of *H. italicum* essential oils have different biological properties and consequently are applied in various industries (Hladnik et al., 2023). Our research has shown that there are three geographically structured chemotypes along the eastern Adriatic coast: Chemotype A (northern Adriatic), Chemotype B (central Adriatic) and Chemotype C (southern Adriatic). Chemotype A, with a high content of neryl acetate and a low content of *ar*-curcumene, is most similar to the chemotype of immortelle populations from Corsica, which are characterised by predominantly oxygenated compounds (neryl acetate, neryl propionate, aliphatic ketones and β-diketones) and a low content of hydrocarbons (limonene, γ-curcumene, α-curcumene) (Bianchini et al., 2001). Chemotype C, which has a high content of *ar*-curcumene and a low content of neryl acetate, is more similar to the chemotypes from Italy (Bianchini et al., 2003). According to review on *H. italicum* essential oil composition and cluster analysis by Aćimović et al. (2021) there are ten chemotypes of *H. italicum* depending on the main compounds in the essential oil: (1) high neryl-acetate chemotype (50.5 – 83.4%) in Sardinia; (2) moderate neryl acetate chemotype (19.5 – 48.0%) in Italy, Corsica, Montenegro and Croatia; (3) neryl-acetate + *ar*-curcumene (3.9 – 20.3% and 0.8 – 14.5%, respectively) in Italy, Algeria, France, and Croatia; (4) *ar*-curcumene + γ-curcumene (17.9 – 28.6% and 12.0 – 22.0%, respectively) in Croatia; (5) γ-curcumene (13.6 – 27.7%) in Serbia, Montenegro, Italy, and the USA; (6) high α-pinene chemotype (25.2 – 53.5%) in Italy, Portugal, and Croatia; (7) moderate α-pinene (5.6 – 20.0%) in Bulgaria, Croatia, Bosnia and Hercegovina, and Algeria; (8) juniper camphor (25.3 – 45.1%) Sardinia; (9) β-selinene (11.6 – 38.0%), and (10) italidiones chemotype., It should be noted that these chemotypes mentioned above were not all analyzed from the natural populations (e.g. *H. italicum* does not grow naturally in the USA or Serbia), and also in some studies the origin of the plant material is unknown and no subspecies specified. Talic et al. (2021) compared composition of *H. italicum* essential oil from Bosnia and Herzegovina with the other Mediterranean countries and concluded that essential oils from Bosnia and Herzegovina are more similar to those from Adriatic region, Croatia and southeast Italy, and the most different from Tyrrhenian islands’ oils.

In our research, the two most prominent compounds that have separated populations into the three chemotypes: neryl acetate and *ar*-curcumene have significant applications in industry. Nerol and its derivatives such as neryl acetate are used in the perfume and cosmetics industries because they contain a desirable refreshing, fruity-floral, sweet scent for blossom compositions (Surburg and Panten, 2006). Esters of short and medium-chain fatty acids and acyclic monoterpene alcohols are essential as fragrances and flavor substances (Genčić et al., 2022). Neryl acetate improves skin barrier function and moisture retention in age-associated skin conditions (Lemaire et al., 2023). Neryl acetate is also attributed sedative effect, which may help promote relaxation and also antioxidant and anti-inflammatory properties, which may help reduce pain and swelling in the body (Buchbauer et al., 1993). Compound α-curcumene, a natural sesquiterpene has anti-inflammatory and antioxidant properties, and is also used in the food industry (Ansari and Curtis, 1974).

High variability in the chemical composition of other Mediterranean plants was also found. In the study of 25 populations of *S. officinalis* on the eastern Adriatic coast, three chemotypes were identified: cis-thujone, trans-thujone and camphor/β-pinene/borneol/bornyl acetate (Jug-Dujaković et al., 2012). Analysis of the chemical diversity of the essential oils of five wild *Origanum vulgare* populations in Montenegro revealed that in *O. vulgare* subsp. *hirtum* the dominant component was the oxygenated monoterpene carvacrol, while in *O. vulgare* subsp. *vulgare* the sesquiterpene hydrocarbons: germacrene D and β-caryophyllene and the oxygenated monoterpenes: α-terpineol, linalyl acetate, linalool, thymol, terpene-4-ol were abundant (Stešević et al., 2018). In another study, essential oils of *Origanum elongatum* from 30 wild populations in Morocco were analyzed. Four chemotypes were identified: carvacrol, carvacrol/thymol, carvacrol/p-cymene and thymol (Bakha et al., 2020).

### 4.3 Diversity of essential oil composition as influenced by geography, climate and genetics

The biochemical diversity of the *H. italicum* populations in our study can largely be explained by the geographical distance between the populations. Morone-Fortunato et al. (2010) also found that genotypes of *H. italicum* from different locations in Italy show variability in essential oil composition due to their geographical origin. In our study, a significant but lower correlation was found between the biochemical and genetic distance, which is consistent with the studies on *H. italicum* from Italy and Corsica, where a correlation was found between the genetic and chemical diversity of the populations studied (Morone-Fortunato et al., 2010). The same results were obtained by Angioni et al. (2003) and Melito et al. (2013) in the studies of *H. italicum* from Sardinia, which confirmed that the biochemical diversity of these populations is strongly influenced by the genotype. In contrast, the study of natural populations of Dalmatian sage (Jug-Dujaković et al., 2012) and Dalmatian pyrethrum (Grdiša et al., 2013) sampled along the eastern Adriatic coast found no correlation between genetic and biochemical distance.

The secondary metabolites of plants are also strongly influenced by bioclimatic factors which means that in various ecological conditions, the same plant would accumulate different metabolites (Zeng et al., 2022). Our research area – Eastern Adriatic region is situated in the northernmost part of the Mediterranean Sea and experiences hot, dry summers and moderate, wet winters, which are typical of the Mediterranean climate. In these conditions, we aimed to further investigate the effects of the bioclimatic variables of this native environment on the essential oil composition of *H. italicum* since the results of our study showed that 22.4% of the biochemical differentiation between the studied populations could be explained by bioclimatic factors. Among the 19 bioclimatic variables used from the WordClim database, bioclimatic variables BIO14 Precipitation of driest month and BIO15 Precipitation seasonality were the most informative variables. Nerol, neryl acetate, and neryl propionate were detected at higher levels in the northern Adriatic populations (P01, P02, P03, and P04), and their synthesis was related to increased precipitation during the warmer months. In contrast, uneven precipitation patterns, particularly a lack of rain in the summer, were found to be detrimental. Meanwhile, the compounds ar-curcumene and β-selinene, which predominated in southern Adriatic populations (P12, P13, P15, P16, P17, P18), exhibited a preference for drier conditions and responded favorably to irregular rainfall distribution throughout the year, indicating a tolerance for water stress characterized by significant fluctuations in precipitation. The formation of secondary metabolites is a complex and not completely investigated process. In some plants, stress will affect the growth and development of the plants but often in medicinal and aromatic plants abiotic stress can enhance accumulation of specific chemical groups of secondary metabolites (Karuppusamy, 2009; Mahajan et al., 2020). In the study of Bettaieb et al. (2009) and Radwan et al. (2017) they concluded that drought stress enhanced the concentration of three most predominant cyclic monoterpenes cineole, camphor, and β thujone in *Salvia officinalis* L. During drought stress, *Hypericum brasiliense* Choisy showed a substantial rise in the concentration of betulinic acid and other phenolic compounds (De Abreu and Mazzafera, 2005). *Melissa officinalis* L. and *Nepeta cataria* L. were reported to have lower total terpenoids during drought stress (Manukyan, 2011). Drought stress can also induce negative effects such as reduction in yield and yield components in *Salvia Sclarea L.* (Asadi et al., 2012). In the study by Varga et al. (2021) on Dalmatian pyrethrum, an insecticidal plant species whose distribution area overlaps with that of immortelle along the eastern Adriatic coast, Precipitation seasonality (BIO15) correlated with the total pyrethrin, cinerin I and jasmolin II content, while the Precipitation of driest month (BIO14) correlated with the total pyrethrin and pyrethrin I content. In another investigation on Dalmatian pyrethrum (Grdiša et al., 2024) the synthesis of pyrethrin I was associated with wide range of temperature fluctuations throughout the year, as well as higher temperatures (as indicated from the variables BIO04 Temperature seasonality, BIO07 Temperature annual range and BIO02 Mean diurnal range) and drought stress during the summer months (as inferred from the variables BIO18 Precipitation of the warmest quarter). Zhou et al. (2021b) investigated the relationship between environmental variables and flavonoid metabolites in *A. squarrosum* and found that quercetin, tricin, and rutin were strongly positively correlated with latitude, longitude, and precipitation gradients, such as annual precipitation, precipitation of the warmest quarter, precipitation of wettest quarter, and precipitation of the wettest month.

In this study, we have demonstrated the high variability of secondary metabolites - essential oils of *H. italicum* along the eastern Adriatic coast, which has adapted genetically slowly from southeast to northwest due to certain bioclimatic variables of the native environment. The eastern Adriatic coast is suitable for analyzing the differences between phenotypic plasticity and local adaptation, as the species genetically adapt along the temperature and precipitation gradient of the eastern Adriatic, which we have demonstrated in previous studies on the genetic analysis of immortelle (Ninčević et al., 2021) and which was also found in the studies on sage (Jug-Dujaković et al., 2012) and Dalmatian pyrethrum (Grdiša et al., 2013). Natural populations of *H. italicum* are important plant genetic resources that can contribute to maintaining sustainable biodiversity and development of new cultivars with desirable and adaptive traits. As the Mediterranean region is highly vulnerable to climate change (Nardini et al., 2014), future projections for the global climate are important for making plans for the sustainability of different economic sectors, including agriculture. In projections of the far future for the Adriatic region, the AdriSC climate model was used to predict a range of extreme climate changes, including intensified heat waves, strong land-sea contrasts in the atmosphere, extreme rainfall, and droughts (Tojčić et al., 2024) which can pose a significant hazard to ecosystems. For a species to persist in harsh environments, genetic adaptation might be needed even while phenotypic plasticity may help short-term adaptation to environmental changes (Richter et al., 2012).

Future research should be extended to other abiotic factors (soil type, salinity, heavy metal content, nutrient deficiencies, UV radiation), affecting the intraspecific variability of *H. italicum*, and sampling should be extended from the eastern Adriatic coast to other native environments. Only deeper understanding of diversity among plant species and mechanisms involved in adaptation to climate change can help us navigate through preservation of ecosystems and improving plant production by developing resistant cultivars.

## 5 Conclusion

This research revealed a high variation of *H. italicum* essential oil from populations distributed along the eastern Adriatic coast, attributing this variability to genetic adaptability rather than phenotypic plasticity, as evidenced by data obtained from field trials. Populations were grouped into three chemotypes based on the presence of neryl acetate and *ar*-curcumene compounds, with Northern Adriatic populations exhibiting higher levels of neryl acetate and southern populations showing dominance in *ar*-curcumene. The observed variability in compound production suggests a direct response to environmental differences in the populations’ native environment, supported by correlations with bioclimatic variables. Higher precipitation during the warmer months was found to potentially enhance the presence of neryl acetate, while uneven precipitation patterns, particularly summer deficits, were identified as unfavorable for this compound. In contrast, drier conditions and irregular rainfall distribution throughout the year were beneficial for *ar*-curcumene. These findings hold the potential for selecting individuals with desirable traits for future breeding programs and commercial cultivars of *H. italicum.* Recognizing the urgency posed by climate change on both ecology and agriculture, the cultivation of elite germplasms tailored to local adaptation emerges as a crucial strategy.

## Data Availability

The raw data supporting the conclusions of this article will be made available by the authors, without undue reservation.
