# Supplementary material for: Phenotypic plasticity vs. local genetic adaptation: essential oil diversity of natural immortelle (Helichrysum italicum (Roth.) G.Don) populations along eastern Adriatic coast
Source: Front Plant Sci. 2025 Feb 5;16:1467421. doi: 10.3389/fpls.2025.1467421 (PMC11836004; doi:10.3389/fpls.2025.1467421)
Supplement: Supplementary file 1 [file Table1.docx]

**Table S1.** **Sampling sites of 18 natural populations of *Helichrysum italicum***

| No. | Accession No.^a^ | Population | Latitude (N)^b^ | Longitude (E)^b^ | Elevation (m a.s.l.) |
| --- | --- | --- | --- | --- | --- |
| P01 | MAP02687 | Krk | 45.23 | 14.58 | 36 |
| P02 | MAP02686 | Cres | 44.83 | 14.42 | 248 |
| P03 | MAP02685 | Lošinj | 44.59 | 14.41 | 73 |
| P04 | MAP02684 | Rab | 44.70 | 14.86 | 44 |
| P05 | MAP02683 | Pag (Zrće) | 44.53 | 14.92 | 18 |
| P06 | MAP02682 | Pag (Miškovići) | 44.33 | 15.24 | 42 |
| P07 | MAP02688 | Obrovac | 44.22 | 15.67 | 137 |
| P08 | MAP02672 | Benkovac | 44.05 | 15.81 | 203 |
| P09 | MAP02673 | Kistanje | 44.02 | 15.89 | 302 |
| P10 | MAP02674 | Unešić | 43.75 | 16.16 | 390 |
| P11 | MAP02675 | Seget | 43.61 | 16.17 | 426 |
| P12 | MAP02677 | Brač | 43.36 | 16.48 | 290 |
| P13 | MAP02679 | Hvar | 43.14 | 16.74 | 361 |
| P14 | MAP02676 | Sinj | 43.67 | 16.65 | 343 |
| P15 | MAP02678 | Omiš | 43.40 | 16.85 | 116 |
| P16 | MAP02680 | Živogošće | 43.18 | 17.20 | 125 |
| P17 | MAP02681 | Slano | 42.83 | 17.82 | 338 |
| P18 | MAP02689 | Cavtat | 42.59 | 18.26 | 525 |

^a^ Accession number from the Collection of Medicinal and Aromatic Plants, as available at the CPGRD (<https://cpgrd.hapih.hr>).

^b^ N - North; E - East; Coordinates are in degree decimal format
