# Supplementary material for: Phenotypic plasticity vs. local genetic adaptation: essential oil diversity of natural immortelle (Helichrysum italicum (Roth.) G.Don) populations along eastern Adriatic coast
Source: Front Plant Sci. 2025 Feb 5;16:1467421. doi: 10.3389/fpls.2025.1467421 (PMC11836004; doi:10.3389/fpls.2025.1467421)
Supplement: Supplementary file 3 [file Table3.docx]

**Table S3. Descriptive statistics of the 18 most abundant essential oil compounds of *H. italicum* calculated based on all samples (18 populations × 2 experimental locations × 2 replicates)**

| Parameter | C03 | C13 | C21 | C35 | C42 | C48 | C49 | C50 | C51 | C56 | C57 | C58 | C59 | C62 | C67 | C72 | C73 | C80 |
| --- | --- | --- | --- | --- | --- | --- | --- | --- | --- | --- | --- | --- | --- | --- | --- | --- | --- | --- |
|  | α-pinene | limonene | linalool | nerol | neryl-acetate | trans- caryophyllene | trans- α-bergamotene | italidione I | neryl propionate | γ-curcumene | *ar*-curcumene | β-selinene | δ- selinene | β-curcumene | neryl isovalerate | eudesm-5-en-11-ol | italidione III | β-eudesmol |
| Average (%) | 5.766 | 2.490 | 2.402 | 2.484 | 16.222 | 3.979 | 1.381 | 6.040 | 2.963 | 2.620 | 9.734 | 9.083 | 3.891 | 0.386 | 2.676 | 2.536 | 0.773 | 2.802 |
| StDev | 3.246 | 1.428 | 1.167 | 1.326 | 7.998 | 1.492 | 2.043 | 3.122 | 1.853 | 1.450 | 3.763 | 3.075 | 1.597 | 0.768 | 1.940 | 1.851 | 1.453 | 1.161 |
| CV | 56.303 | 57.348 | 48.582 | 53.360 | 49.305 | 37.502 | 147.988 | 51.684 | 62.525 | 55.351 | 38.661 | 33.857 | 41.050 | 198.941 | 72.480 | 72.960 | 188.085 | 41.439 |
| Min | 0.000 | 0.069 | 0.850 | 0.647 | 3.052 | 1.100 | 0.216 | 0.795 | 0.000 | 0.736 | 0.000 | 4.954 | 1.515 | 0.000 | 0.000 | 0.000 | 0.000 | 0.532 |
| Max | 18.137 | 8.763 | 7.184 | 5.846 | 32.581 | 9.375 | 10.429 | 15.941 | 7.306 | 7.692 | 18.882 | 16.974 | 11.445 | 6.254 | 6.603 | 7.293 | 9.145 | 6.410 |

StDev - standard deviation; CV - coefficient of variation; Min - minimum value; Max - maximum value
