# Supplementary material for: Phenotypic plasticity vs. local genetic adaptation: essential oil diversity of natural immortelle (Helichrysum italicum (Roth.) G.Don) populations along eastern Adriatic coast
Source: Front Plant Sci. 2025 Feb 5;16:1467421. doi: 10.3389/fpls.2025.1467421 (PMC11836004; doi:10.3389/fpls.2025.1467421)
Supplement: Supplementary file 4 [file Table4.docx]

Table S4. Correlations between the 18 main *H. italicum* essential oil compounds

| Compound | C03 | C13 | C21 | C35 | C42 | C48 | C49 | C50 | C51 | C56 | C57 | C58 | C59 | C62 | C67 | C72 | C73 | C80 |
| --- | --- | --- | --- | --- | --- | --- | --- | --- | --- | --- | --- | --- | --- | --- | --- | --- | --- | --- |
| C03 |  | ns | ns | ns | ns | * | ns | ns | ns | ns | ns | ns | ns | ns | ns | ns | ns | ns |
| C13 | 0.356 |  | ns | ns | ns | ns | ns | ns | ns | ns | ns | ns | ns | ns | ns | ns | ns | ns |
| C21 | -0.187 | -0.177 |  | ns | ns | ns | ** | ns | *** | ns | ns | ns | ns | ns | ns | ns | ns | ns |
| C35 | -0.285 | -0.157 | 0.444 |  | *** | ns | ns | * | *** | ns | *** | *** | *** | ns | *** | ns | * | ns |
| C42 | -0.231 | -0.002 | 0.205 | 0.866 |  | ns | ns | ** | ** | ns | *** | *** | *** | ns | *** | ns | * | ns |
| C48 | 0.502 | 0.080 | 0.381 | -0.244 | -0.324 |  | ns | ns | ns | ns | ns | ns | ns | ns | ns | ns | ns | ns |
| C49 | 0.295 | 0.142 | -0.593 | -0.377 | -0.234 | -0.302 |  | ns | * | ns | ns | ns | ns | ns | ns | * | ns | ns |
| C50 | -0.398 | -0.279 | -0.030 | -0.469 | -0.602 | 0.009 | -0.243 |  | ns | ns | * | ** | * | ns | ns | ns | ** | ns |
| C51 | -0.303 | -0.211 | 0.736 | 0.786 | 0.675 | 0.012 | -0.576 | -0.272 |  | ns | *** | * | ns | ns | ** | ns | ns | ns |
| C56 | 0.318 | -0.159 | 0.130 | 0.467 | 0.334 | 0.095 | -0.321 | -0.370 | 0.276 |  | ns | * | ** | ns | ns | ns | ns | ns |
| C57 | 0.207 | -0.155 | -0.429 | -0.849 | -0.874 | 0.141 | 0.412 | 0.543 | -0.761 | -0.357 |  | *** | ns | ns | ** | ns | ns | ns |
| C58 | 0.060 | 0.024 | -0.216 | -0.845 | -0.891 | 0.265 | 0.247 | 0.610 | -0.565 | -0.493 | 0.821 |  | *** | ns | ** | ns | * | ns |
| C59 | -0.144 | 0.208 | -0.125 | -0.767 | -0.746 | 0.215 | 0.128 | 0.517 | -0.466 | -0.630 | 0.567 | 0.862 |  | ns | * | ns | ns | ns |
| C62 | -0.452 | -0.251 | -0.381 | -0.355 | -0.233 | -0.354 | 0.137 | 0.431 | -0.288 | -0.423 | 0.252 | 0.219 | 0.434 |  | ns | ns | ns | ns |
| C67 | -0.416 | -0.104 | 0.276 | 0.786 | 0.772 | -0.149 | -0.442 | -0.266 | 0.669 | 0.137 | -0.694 | -0.600 | -0.507 | -0.241 |  | ns | ns | ns |
| C72 | -0.112 | -0.028 | -0.285 | -0.147 | -0.176 | -0.395 | 0.474 | -0.143 | -0.394 | -0.180 | 0.095 | 0.093 | 0.211 | 0.251 | -0.266 |  | ns | * |
| C73 | 0.010 | 0.170 | -0.262 | -0.540 | -0.521 | -0.072 | -0.022 | 0.625 | -0.438 | -0.346 | 0.452 | 0.524 | 0.371 | 0.095 | -0.339 | 0.073 |  | ns |
| C80 | 0.188 | 0.398 | -0.133 | -0.112 | -0.202 | 0.095 | 0.156 | -0.117 | -0.405 | 0.143 | 0.009 | 0.167 | 0.189 | -0.322 | -0.253 | 0.478 | 0.202 |  |

Below the diagonal - Pearson's correlation coefficient (*r*)

Above the diagonal - sign of significance (*P*) of the correlation coefficient

*** *P* < 0.001, ** 0.001 < *P* < 0.01, * 0.01 < *P* < 0.05, ns *P* > 0.05
