# Supplementary material for: Phenotypic plasticity vs. local genetic adaptation: essential oil diversity of natural immortelle (Helichrysum italicum (Roth.) G.Don) populations along eastern Adriatic coast
Source: Front Plant Sci. 2025 Feb 5;16:1467421. doi: 10.3389/fpls.2025.1467421 (PMC11836004; doi:10.3389/fpls.2025.1467421)
Supplement: Supplementary file 5 [file Table5.docx]

Table S5. Principal component analysis (PCA): Correlations between nine *H. italicum* essential oil compounds and the first three principal components

| Compound | PC1 |  | PC2 |  |
| --- | --- | --- | --- | --- |
| C13 | -0.117 | ns | -0.177 | ns |
| C21 | 0.456 | ns | 0.824 | *** |
| C35 | 0.961 | *** | 0.002 | ns |
| C42 | 0.930 | *** | -0.221 | ns |
| C48 | -0.224 | ns | 0.756 | *** |
| C51 | 0.822 | *** | 0.439 | ns |
| C57 | -0.908 | *** | -0.039 | ns |
| C58 | -0.916 | *** | 0.236 | ns |
| C59 | -0.803 | *** | 0.253 | ns |
| Eigenvalue | 5.044 |  | 1.646 |  |
| % of total variance | 56.04 |  | 18.29 |  |

*** *P* < 0.001; ** 0.001 < *P* < 0.01; * 0.01 < *P* < 0.05; ^ns^ *P* > 0.05
