# Supplementary material for: Phenotypic plasticity vs. local genetic adaptation: essential oil diversity of natural immortelle (Helichrysum italicum (Roth.) G.Don) populations along eastern Adriatic coast
Source: Front Plant Sci. 2025 Feb 5;16:1467421. doi: 10.3389/fpls.2025.1467421 (PMC11836004; doi:10.3389/fpls.2025.1467421)
Supplement: Supplementary file 6 [file Table6.docx]

Table S6. Euclidean distance between 18 immortelle populations calculated on the basis of nine essential oil compounds

| Br. | Population | P01 | P02 | P03 | P04 | P05 | P06 | P07 | P08 | P09 | P10 | P11 | P12 | P13 | P14 | P15 | P16 | P17 |
| --- | --- | --- | --- | --- | --- | --- | --- | --- | --- | --- | --- | --- | --- | --- | --- | --- | --- | --- |
| P01 | Krk |  |  |  |  |  |  |  |  |  |  |  |  |  |  |  |  |  |
| P02 | Cres | 1.600 |  |  |  |  |  |  |  |  |  |  |  |  |  |  |  |  |
| P03 | Lošinj | 8.438 | 8.311 |  |  |  |  |  |  |  |  |  |  |  |  |  |  |  |
| P04 | Rab | 5.905 | 6.347 | 4.776 |  |  |  |  |  |  |  |  |  |  |  |  |  |  |
| P05 | Pag 1 | 5.214 | 6.153 | 9.291 | 5.619 |  |  |  |  |  |  |  |  |  |  |  |  |  |
| P06 | Pag 2 | 6.082 | 7.371 | 12.725 | 9.240 | 5.409 |  |  |  |  |  |  |  |  |  |  |  |  |
| P07 | Obrovac | 8.008 | 9.083 | 15.333 | 11.881 | 7.572 | 3.730 |  |  |  |  |  |  |  |  |  |  |  |
| P08 | Benkovac | 8.680 | 9.839 | 15.644 | 12.067 | 7.644 | 4.614 | 3.242 |  |  |  |  |  |  |  |  |  |  |
| P09 | Kistanje | 6.048 | 7.133 | 12.641 | 8.922 | 4.579 | 3.745 | 4.689 | 4.000 |  |  |  |  |  |  |  |  |  |
| P10 | Unešić | 7.567 | 8.441 | 15.261 | 12.013 | 7.622 | 4.185 | 2.570 | 3.019 | 4.107 |  |  |  |  |  |  |  |  |
| P11 | Seget | 4.991 | 6.029 | 10.550 | 7.549 | 3.724 | 4.527 | 6.242 | 5.731 | 3.057 | 5.603 |  |  |  |  |  |  |  |
| P12 | Brač | 19.153 | 20.095 | 26.529 | 23.204 | 18.825 | 14.174 | 11.845 | 11.849 | 15.160 | 12.041 | 16.883 |  |  |  |  |  |  |
| P13 | Hvar | 18.201 | 18.847 | 26.114 | 23.007 | 18.632 | 14.290 | 11.549 | 11.865 | 14.826 | 11.190 | 16.416 | 4.957 |  |  |  |  |  |
| P14 | Sinj | 5.770 | 6.397 | 13.615 | 10.301 | 6.343 | 4.739 | 4.351 | 5.251 | 4.373 | 3.530 | 5.760 | 14.770 | 13.729 |  |  |  |  |
| P15 | Omiš | 12.281 | 13.119 | 19.961 | 16.429 | 12.439 | 7.670 | 6.342 | 7.488 | 9.210 | 6.426 | 11.245 | 8.567 | 8.589 | 8.115 |  |  |  |
| P16 | Živogošće | 11.989 | 12.839 | 19.473 | 15.862 | 11.105 | 7.451 | 5.415 | 5.391 | 7.247 | 4.882 | 9.473 | 8.936 | 8.561 | 7.413 | 4.629 |  |  |
| P17 | Slano | 17.932 | 18.728 | 25.514 | 22.476 | 18.188 | 13.530 | 11.087 | 11.353 | 14.431 | 10.937 | 15.975 | 2.979 | 3.110 | 13.675 | 7.976 | 8.318 |  |
| P18 | Cavtat | 20.219 | 21.083 | 27.694 | 24.252 | 19.688 | 15.616 | 12.837 | 12.439 | 15.842 | 12.862 | 17.684 | 3.690 | 5.093 | 15.493 | 10.489 | 9.621 | 4.836 |
