# Supplementary material for: Phenotypic plasticity vs. local genetic adaptation: essential oil diversity of natural immortelle (Helichrysum italicum (Roth.) G.Don) populations along eastern Adriatic coast
Source: Front Plant Sci. 2025 Feb 5;16:1467421. doi: 10.3389/fpls.2025.1467421 (PMC11836004; doi:10.3389/fpls.2025.1467421)
Supplement: Supplementary file 7 [file Table7.docx]

Table S7. The most informative compounds for the discrimination of three chemotypes of immortelle based on stepwise discrimination analysis

|  | Compound | Partial *R^2^* | *F* | *P*(F) |
| --- | --- | --- | --- | --- |
| C35 | nerol | 0.881 | 55.26 | < 0.0001 |
| C51 | neryl propionate | 0.587 | 9.94 | 0.002 |
| C48 | trans-caryophyllene | 0.471 | 5.78 | 0.016 |
| C58 | β-selinene | 0.412 | 4.20 | 0.041 |
