# Supplementary material for: Phenotypic plasticity vs. local genetic adaptation: essential oil diversity of natural immortelle (Helichrysum italicum (Roth.) G.Don) populations along eastern Adriatic coast
Source: Front Plant Sci. 2025 Feb 5;16:1467421. doi: 10.3389/fpls.2025.1467421 (PMC11836004; doi:10.3389/fpls.2025.1467421)
Supplement: Supplementary file 8 [file Table8.docx]

Table S8. Correlation between sampling site (latitude and longitude) and the 18 main essential oil compounds

| Compound | Latitude (N) |  | Longitude (E) |  |
| --- | --- | --- | --- | --- |
| C03 | -0.345 | ns | 0.334 | ns |
| C13 | 0.030 | ns | 0.035 | ns |
| C21 | 0.349 | ns | -0.392 | ns |
| C35 | 0.824 | *** | -0.805 | *** |
| C42 | 0.855 | *** | -0.866 | *** |
| C48 | -0.342 | ns | 0.271 | ns |
| C49 | -0.186 | ns | 0.180 | ns |
| C50 | -0.631 | ** | 0.616 | ** |
| C51 | 0.680 | ** | -0.689 | ** |
| C56 | 0.188 | ns | -0.233 | ns |
| C57 | -0.845 | *** | 0.824 | *** |
| C58 | -0.768 | *** | 0.741 | *** |
| C59 | -0.486 | * | 0.491 | * |
| C62 | -0.085 | ns | 0.116 | ns |
| C67 | 0.633 | ** | -0.622 | ** |
| C72 | 0.082 | ns | -0.003 | ns |
| C73 | -0.632 | ** | 0.684 | ** |
| C80 | -0.122 | ns | 0.145 | ns |

ns non-significant; *significant at P < 0.05; **significant at P < 0.01; ***significant at P < 0.001
